# Supplementary material for: Demonstrating Biological Fate of Nanoparticle-Loaded Dissolving Microneedles with Aggregation-Caused Quenching Probes: Influence of Application Sites
Source: Pharmaceutics. 2023 Jan 3;15(1):169. doi: 10.3390/pharmaceutics15010169 (PMC9867466; doi:10.3390/pharmaceutics15010169)
Supplement: Supplementary file 1 [file pharmaceutics-15-00169-s001.zip › pharmaceutics-2091679-supplementary.pdf]

pharmaceutics

MDPI

Supplementary materials

Demonstrating Biological Fate of Nanoparticle-Loaded Dissolving Microneedles with Aggregation-Caused Quenching Probes: Influence of Application Sites

Yanping Fu [1], Chaonan Shi [1], Xiaodie Li [1], Ting Wen [2], Qiaoli Wu [3], Antian Zhang [1], Ping Hu [1], Chuanbin Wu [1], Xin Pan [2], Zhengwei Huang [1,*], and Guilan Quan [1,*]

[1] College of Pharmacy, Jinan University, Guangzhou 510632, China
[2] School of Pharmaceutical Sciences, Sun Yat-sen University, Guangzhou 510006, China
[3] The Fourth Affiliated Hospital of Guangzhou Medical University, Guangzhou 511300, China
[*] Correspondence: huangzhengw@jnu.edu.cn (Z. Huang), quanguilan@jnu.edu.cn (G. Quan)

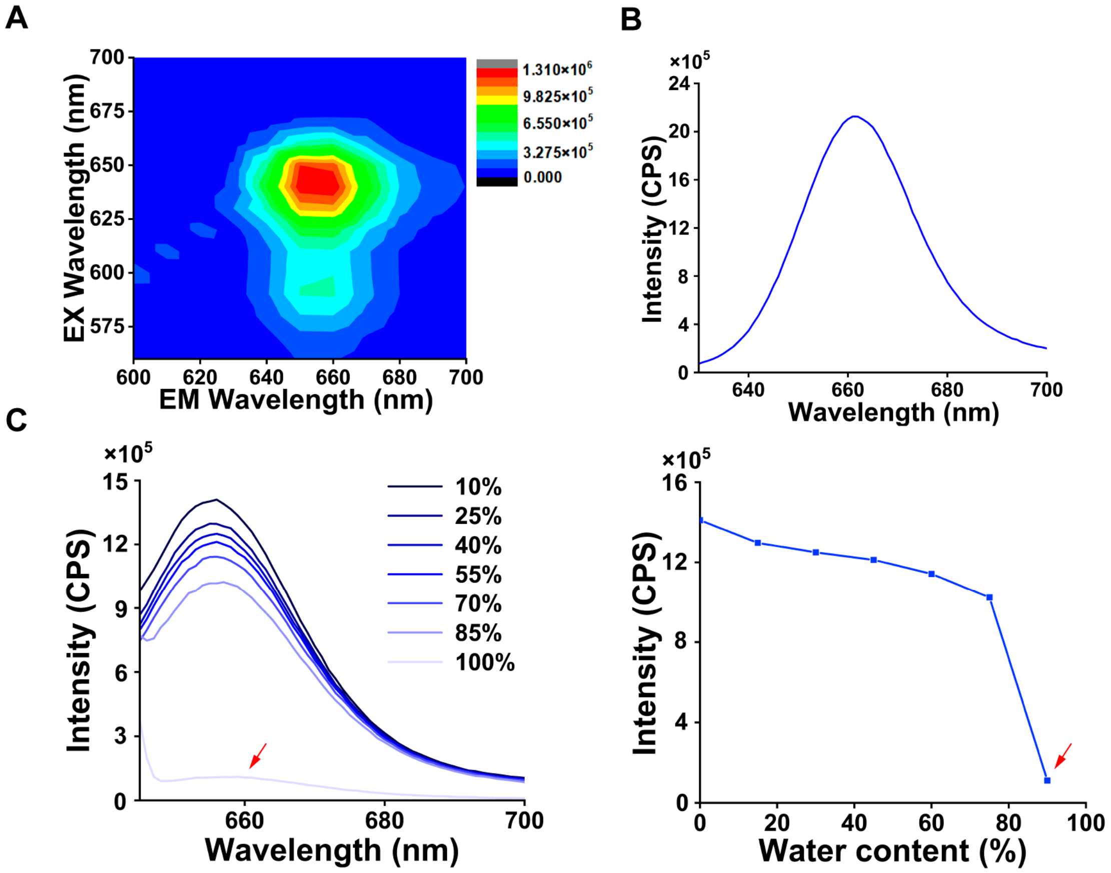

Figure S1. (**A**) Fluorescence emission contour map of P4 probes. (**B**) Fluorescence emission spectrum of P4 probes. (**C**) Fluorescence emission spectra (left) and peak intensity transition (right) of P4 probes in water-acetonitrile co-solvent with 10-100% (v/v) water content.

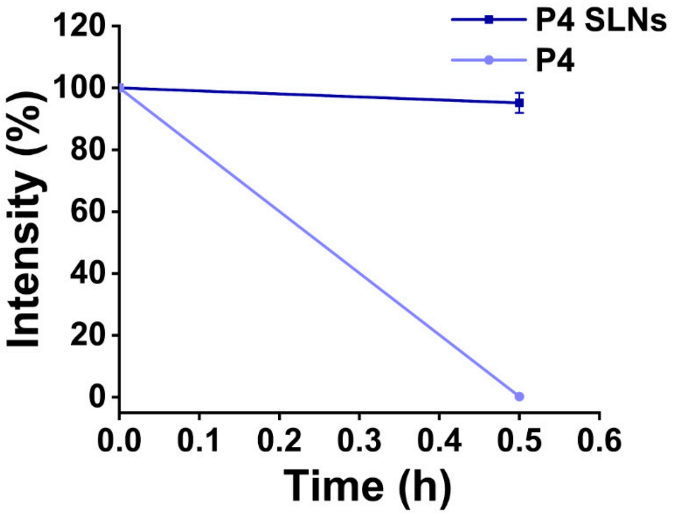

Figure S2. Fluorescence intensity of P4 and P4 SLNs incubated with phosphate-buffered saline solution for 0.5 h, respectively ($n = 3$). Data are expressed as mean ± SD.

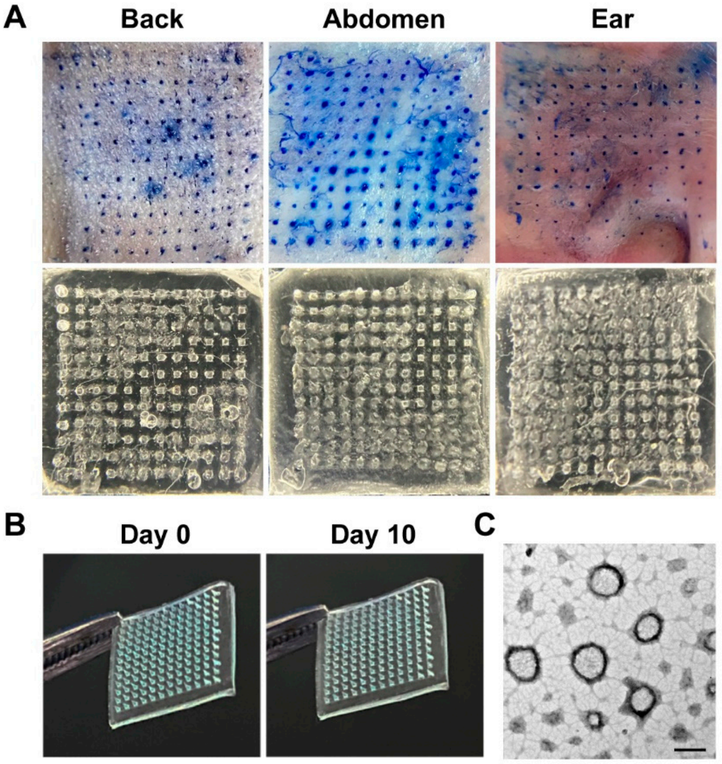

Figure S3. (**A**) The photographs of the skin by trypan blue staining (up) and the base of DMNs (down) after inserting with P4 SLNs@DMNs. (**B**) The appearance of P4 SLNs@DMNs before and after 10 days. (**C**) TEM image of dissolved P4 SLNs from P4 SLNs@DMNs (scale bar: 200 nm).

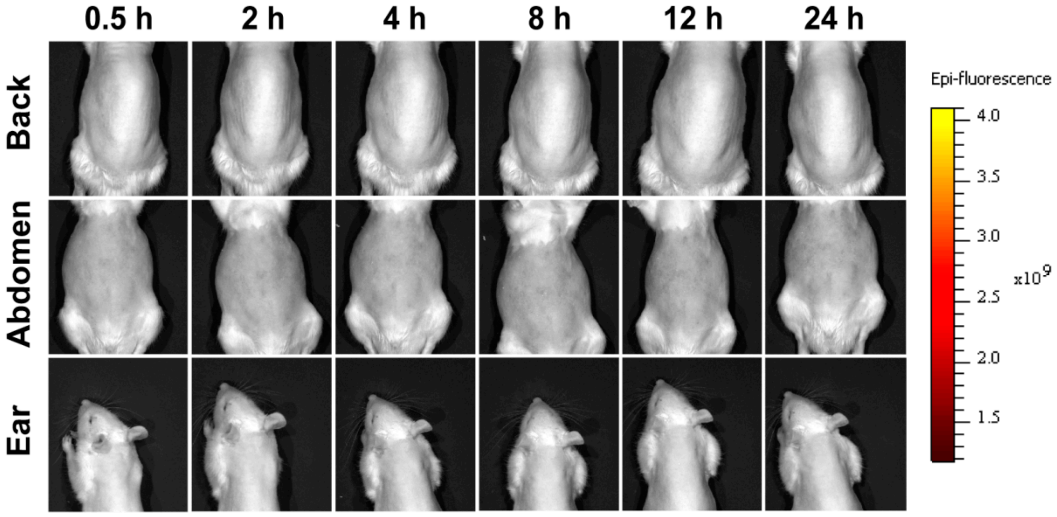

Figure S4. *In vivo* live imaging. Representative live images of rats without any treatment.

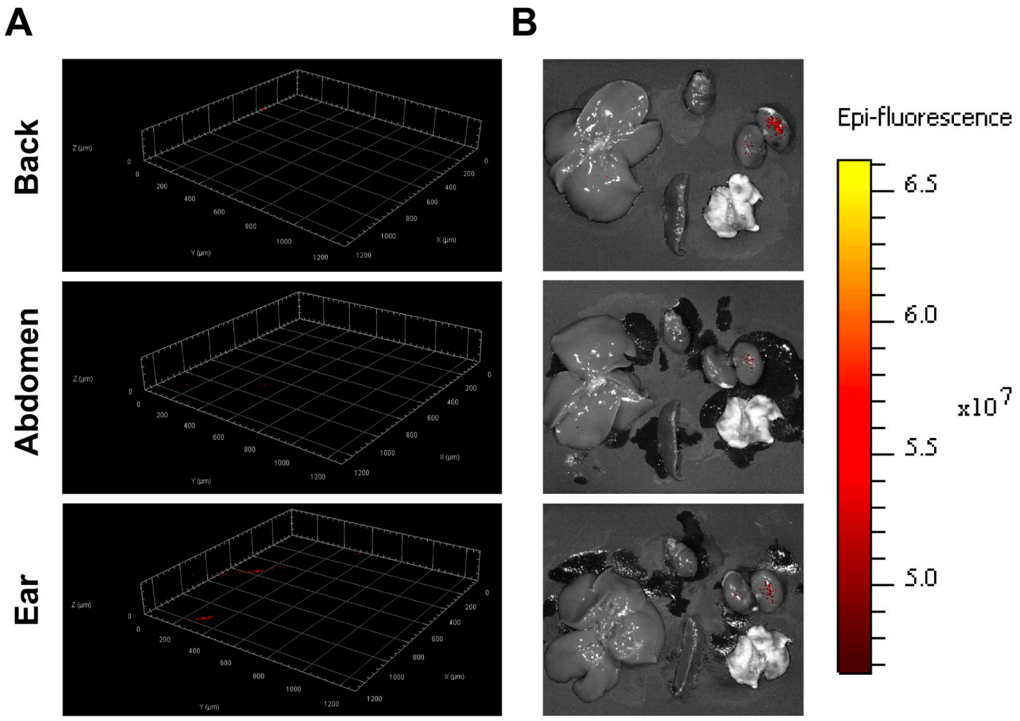

Figure S5. *Ex vivo* imaging. **(A)** Representative CLSM 3D reconstruction images of the skin of back, abdomen, and ear without any treatment at 4 h. **(B)** Representative fluorescent images of major organs without any treatment at 4 h.
